# Analysis of the Applicability of Accelerated Conditioning Protocols in Concrete Beams Reinforced with Steel and GFRP: Effects of Chloride Exposure

**DOI:** 10.3390/polym17172423

**Published:** 2025-09-07

**Authors:** Amanda Duarte Escobal Mazzú, Gláucia Maria Dalfré

**Affiliations:** Graduate Program in Civil Engineering (PPGECiv), Department of Civil Engineering (DECiv), Federal University of Sao Carlos (UFSCar), Sao Carlos 13565-905, Brazil; amandadescobal@hotmail.com

**Keywords:** GFRP, steel, corrosion, chloride, concrete, beams, reinforcement

## Abstract

The durability of Fiber-Reinforced Polymer (FRP) bars is typically evaluated using accelerated conditioning protocols (ACP), which are applied to bar samples, either directly exposed or embedded in small concrete specimens, under aggressive environmental conditions. Thus, this study investigates the applicability of the ACPs recommended by ACI440.9R (2015), from the American Concrete Institute, to assess the potential effects of chloride exposure on reinforced concrete beams. Twelve beams—six reinforced with steel and six with Glass Fiber-Reinforced Polymer (GFRP)—were tested under two scenarios: (1) a reference condition, with beams stored for 1000 h in a controlled laboratory environment, and (2) a conditioned condition, where beams were immersed in a 3.5% NaCl solution at 50 ± 3 °C for 1000 h prior to beam casting. After, the beams were evaluated through three-point bending tests, focusing on load–deflection behavior, failure modes, crack patterns, and strain distribution in concrete and reinforcement. The results indicated that chloride exposure adversely affected both steel and GFRP-reinforced beams. Steel-reinforced concrete beams exhibited a 12% reduction in load-bearing capacity due to steel corrosion, while the GFRP-reinforced concrete beams showed a 10% reduction in load-bearing capacity due to water absorption by the GFRP.

## 1. Introduction

Traditional reinforced concrete structures may experience corrosion in steel reinforcement when exposed to aggressive environments, mainly due to carbonation or chloride attack. The primary cause of concrete structures deterioration is steel reinforcement corrosion, which leads to significant economic losses due to maintenance and the reduced performance of structural components [1,2,3,4,5,6,7,8]. Addressing the issue of steel reinforcement corrosion in aggressive environments is crucial for ensuring load capacity and durability of concrete structures [9]. As a result, alternative materials have been explored, with Fiber-Reinforced Polymers (FRP) emerging as a promising substitute for steel reinforcement in aggressive conditions [3,9,10,11,12,13]. Research and development in FRP composites have significantly increased over the past two decades. While this is not a new research area, there is still no consensus among researchers worldwide regarding the reliability and durability of FRPs [14].

FRP is a composite material that combines continuous fibers and a polymeric matrix (resin). The most used fibers are carbon, glass, aramid and basalt [6,15,16]. Among these fibers, glass emerges as a preferred choice for concrete reinforcement due to its chemical resistance, high strength-weight ratio, corrosion resistance, cost-effectiveness, and environmental sustainability [17,18,19,20,21]. The polymeric matrix is responsible for bonding the fibers and distributing the stresses imposed on the composite among individual fibers while protecting against abrasion, impact, and environmental conditions that could affect the durability of the FRP [22]. The resin used to produce the FRP is critical for achieving the desired mechanical properties and durability characteristics.

FRP is corrosion-resistant, exhibits high tensile strength, is lightweight, and transparent to electromagnetic fields [23,24,25,26,27,28]. Despite its advantages, FRP is susceptible to degradation in tensile properties when exposed to some aggressive conditions [29,30,31]. A significant reduction in the tensile strength of GFRP (Glass Fiber-Reinforced Polymer) bars can be observed due to polymer degradation, fiber-matrix debonding, or fiber corrosion [7,32]. In recent years, numerous studies have been conducted to investigate the durability of GFRP bars under various environmental conditions. The factors that contribute most to the degradation of GFRP are moisture absorption, alkaline environments, and temperature [3]. Moisture penetrates the polymeric matrix through solution flow in the micro gaps between polymer chains and via capillary transport across microcracks at the fiber/matrix interface or microcracks formed during manufacturing [33]. The polymeric matrix degrades through a hydrolytic reaction, in which water molecules interact with the ester groups in the polymer (such as polyester or vinyl ester resin), leading to irreversible chemical changes that reduce both toughness and fracture strain [27]. Consequently, the matrix undergoes plasticization, which induces reductions in strength and glass transition temperature [3]. One significant issue with using GFRP as reinforcement is its sensitivity to alkaline attacks due to the surrounding concrete and intrinsically aggressive environment. The alkalinity of the solution contained in concrete pores is primarily responsible for the degradation of GFRP bars [34]. This degradation occurs as hydroxyl ions (OH-) penetrate the polymeric matrix and react with resin ester, forming alcohol and carboxylic acid salt in a process known as hydrolysis [35]. As hydroxyl ions diffuse, silica bonds are broken, leading to a loss of weight and strength in the glass fibers [36].

Understanding the long-term degradation of GFRP materials under harsh environments is essential for achieving long-term durable performance over 75 years or more [37]. Since the use of FRP as reinforcement in concrete structures became widespread in the 1980s, and virtually all concrete structures with this type of reinforcement have been designed for a service life of over 40 years, most available data regarding their actual long-term performance is limited [26]. The durability of FRP is closely related to its long-term performance, which can be assessed over 20 to 50 years [38]. Most studies subjected GFRP bars to accelerated conditioning for 3 to 36 months at elevated temperatures [5]. One approach to identifying vulnerabilities in the long-term behavior of FRP involves applying accelerated conditioning protocols (ACPs), such as those outlined in ACI 440.9R [31]. Using these ACPs, combined with the characterization of the mechanical properties over time, allows for the assessment of material durability. Most studies have utilized accelerated conditioning techniques, where FRP bars are immersed in aggressive solutions at elevated temperatures to evaluate their physical and mechanical integrity over time [3,10,15,19,39,40]. However, ACPs are applied to FRP bar samples and not to concrete elements reinforced with FRP, disregarding the interaction between the concrete and the embedded FRP reinforcement. This approach tends to be more severe than the material degradation observed in service [3,19,32,41]. In real-world scenarios, the FRP bar will not be directly exposed to the aggressiveness of the environment. Chen et al. [32] investigated the durability of non-protected and concrete-protected GFRP bars, manufactured with E-glass fibers and vinyl ester resin, under accelerated aging environments. After 2 months of exposure to an environment simulating the pore solution of concrete at 60 °C, the tensile strength retention of the GFRP bars was 52%. For the concrete-protected GFRP bars, the tensile strength retention was 61% after 3 months of exposure. The authors concluded that simulated environments were more aggressive to non-protected GFRP bars than those embedded in concrete. Therefore, evaluating the behavior of bars embedded in concrete provides a more realistic expectation of the degradation that bars may undergo when used as reinforcement in concrete structures [15].

According to *Bulletin* 40 [29], from the International Federation for Structural Concrete (fib), distinguishing between degradation caused by chloride attack, moisture diffusion, and alkalinity remains challenging. GFRP bars exposed to the combined effects of chlorides and moisture in concrete have demonstrated reductions in strength and stiffness by approximately 50%. The deterioration of FRP materials may not solely be attributable to chloride attack, but also to alkaline attack or matrix plasticization induced by moisture action. Robert, Cousin, and Benmokrane [42] conducted accelerated tests to assess the durability of GFRP bars, manufactured with E-glass and vinyl ester resin, when embedded in concrete. After 8 months of exposure to tap water at 50 °C, the tensile strength retention of the samples was 85%. In another study, Robert and Benmokrane [43] subjected GFRP bars (E-glass and vinyl ester resin) embedded in concrete to a 3% saline solution (NaCl) at 23 °C, 40 °C, and 50 °C for 60, 120, 210, and 365 days. After 365 days of exposure to the saline solution at 50 °C, the tensile strength retention was 89%. Additionally, GFRP bars embedded in concrete were exposed to a 3 % saline solution (NaCl) and tap water at 70 °C for 120 days. The results did not reveal significant differences in the durability of GFRP bars immersed in either tap water or saline solution, thereby confirming the long-term durability of GFRP bars under saline solution exposure. The authors predicted that, even after a service life of 100 years, the tensile strength retention of the studied bars would remain at 70% and 77% for average annual temperatures of 50 °C (average annual temperature in the Middle East and other hot regions) and 10 °C (average temperature of northern regions), respectively. Almusallam et al. [44] investigated the effects of different environmental conditions on the properties of GFRP bars (E-glass and vinyl ester resin) embedded in concrete over 6, 12, and 18 months. The residual tensile strength testing results showed that the specimens conditioned in tap water at 50 °C experienced more pronounced degradation than those exposed to seawater at the same temperature. The tensile strength ranged from 83% to 76% for exposure to tap water, and between 86 % and 84% for exposure to seawater. Fergani et al. [2] examined the degradation of GFRP bars (ECR glass and vinyl ester resin) embedded in concrete and conditioned in tap water at 60 °C for up to 9 months, observing tensile strength retentions between 80% and 59%. Jia et al. [4] analyzed the short-term durability of GFRP bars, manufactured with E-glass and vinyl ester resin, embedded in concrete and exposed to various solutions and ambient humidity. The results indicated that the specimens subjected to tap water and saline solution for 4 months exhibited tensile strength retentions of 61.6% and 60.7%, respectively. Morales et al. [26] investigated the durability performance of ECR glass and vinyl ester manufactured GFRP bars embedded in concrete mixed with seawater. The GFRP bars embedded in concrete were exposed to conditions simulating typical field environments in a subtropical region and seawater at 60 °C for 1, 6, 12, and 24 months. After mechanical and physical evaluations, these researchers concluded that environmental conditions influenced the tensile strength of the GFRP bars. Based on an experimental degradation model, the long-term prediction of the tensile strength retention averaged 92% under subtropical region exposure and 72% under seawater at 60 °C. Li et al. [45] investigated the durability of GFRP bars manufactured with vinyl ester resin exposed to seawater, alkaline-saline solution, and seawater immersion within the concrete, all maintained at 60 °C. After Scanning Electron Microscopy (SEM) analysis, the researchers reported no visible damage to the bars after 183 days of exposure to seawater at 60 °C. However, GFRP bars exposed to the alkaline-saline solution and immersed in concrete, as well as those exposed to seawater exhibited varying degrees of fiber-matrix separation after 183 days. This phenomenon was attributed to matrix swelling caused by water absorption. Openings in the polymeric matrix of samples exposed to the alkaline-saline solution were observed, which were attributed to the erosion effect of hydroxyl ions. Pits were also observed in samples immersed in concrete and exposed to seawater, attributed to the hydrolysis reaction. Wang et al. [46] subjected GFRP bars (vinyl ester resin) embedded in concrete mixed with seawater and sea sand to wet-dry cycles in chloride solution for 90, 180, and 270 days. These cycles involved immersion in a 3.5% NaCl solution at a temperature of 40 ± 2 °C for 12 h, followed by drying in an environment with a temperature of 25.2 ± 8.9 °C and humidity of 65.3 ± 16.6% for another 12 h. After the exposure, the bars underwent axial tensile tests, and tensile strength retentions of 81%, 76%, and 76% were observed after 90, 180, and 270 days of exposure, respectively. The researchers note that the degradation of the tensile strength slowed after 90 days of exposure. This reduction in the rate of degradation is attributed to the accumulation of Friedel’s salt, which forms from reactions between chloride ions and tricalcium aluminate in the cement, hindering the penetration of the solution into the GFRP bars, delaying the effects of polymer matrix hydrolysis and GFRP bar degradation. Table 1 provides tensile strength retention results of GFRP bars in concrete.

Previous durability investigations of GFRP reinforcement have primarily applied ACPs at bar level (Table 2), evaluating residual tensile capacity, elastic modulus, or interlaminar shear strength.

This study uniquely contributes to durability research by extending ACI 440.9R [31] accelerated conditioning protocols to the structural scale. Unlike prior work that has been limited to isolated bars, our investigation applies chloride exposure protocols directly to reinforced-concrete beams, providing a benchmark that bridges material-level degradation and structural performance. This approach enables (i) verification of whether ACPs remain sufficiently severe at the full-scale beam, (ii) direct comparison of steel and GFRP reinforcement under identical chloride attack, and (iii) assessment of the conservatism of ACI 440.1R [30] provisions after accelerated degradation.

## 2. Experimental

Twelve reinforced concrete beams were designed according to ACI 318 [47] and ACI 440.1R [30], utilizing steel and GFRP bars for the longitudinal reinforcement, respectively. Six beams were reinforced with 10 mm diameter steel bars, while the remaining six featured 10 mm diameter GFRP bars. Since steel reinforcement corrosion is a primary cause of deterioration in reinforced concrete structures, and corrosion resistance is a key characteristic of FRP, a chloride solution was employed for the accelerated conditioning protocol of the beams. Within each set of six beams, two were stored in a controlled laboratory environment for 45 days (reference), two were exposed to a laboratory environment for 1000 h, and two were immersed in a 3.5% NaCl solution tank with a temperature of 50 ± 3 °C for 1000 h, as detailed in Table 1. The duration of 1000 h of immersion in 3.5% NaCl at 50 ± 3 °C was adopted in accordance with the ACI 440.9R [31] guidelines for accelerated conditioning protocols of FRP reinforcement. This exposure length has been widely used in bar-level studies (e.g., Fergani et al. [2]; Benmokrane et al. [22]; Robert et al. [42]; Robert and Benmokrane [43]) as a standardized baseline to evaluate comparative durability, rather than as a direct simulation of a field service period. In this work, the same protocol was applied at the full-scale beam with the objective of (i) maintaining comparability with existing durability data at the material level, and (ii) ensures comparability with the extensive database of bar-level degradation, while providing insight into whether the severity of ACPs is sufficient to induce measurable differences at the structural scale. So, the study therefore provides a structural-level benchmark that can complement service-life modeling or field-exposure studies in future research.

It is important to note that the 1000 h periods were initiated 45 days after concrete casting, following the testing of the reference beams. As indicated in Table 3, concrete, steel, and GFRP specimens were subjected to the same exposure environments as the beams and tested to determine their mechanical properties.

The beams were designated as Vx_y_z_w, where x is equal to 1 or 2 based on the model repetition, y is equal to LAB for laboratory exposure environment or ACP for accelerated conditioning protocol, z is equal to S for steel reinforcement or GFRP for GFRP reinforcement, and w is equal to 45 days or 1000 h, corresponding to the age of the beams at the time of testing. For instance, V1_ACP_GFRP_1000h represents the first beam reinforced with GFRP tested after 1000 h of exposure to chloride attack. It is important to acknowledge that only two replicas were tested, which is below the minimum generally recommended for more rigorous statistical treatment. This decision was motivated by the consistent behavior typically observed among specimens reinforced or strengthened with FRP systems, as well as by the fact that full-scale structural tests are both costly and time-consuming. Therefore, the results should be interpreted as preliminary trends rather than statistically generalizable outcomes.

### 2.1. Material Properties

Concrete cylinders with a diameter of 100 mm and a height of 200 mm underwent compressive strength and elasticity modulus testing, following the Brazilian Standards ABNT NBR 5739 [48] and ABNT NBR 8522 [49], respectively. Three specimens were used to determine the compressive strength, and two were used for elasticity modulus at each age. The target concrete compressive strength was 30 MPa.

For each age and exposure environment, two steel bars with a length of 60 cm and a diameter of 10 mm underwent tensile tests, according to ABNT NBR ISO 6892-1 [50], to obtain the stress–strain behavior and elasticity modulus. The steel’s nominal yield strength and elasticity modulus were 500 MPa and 200 GPa, respectively.

**Figure 1:**
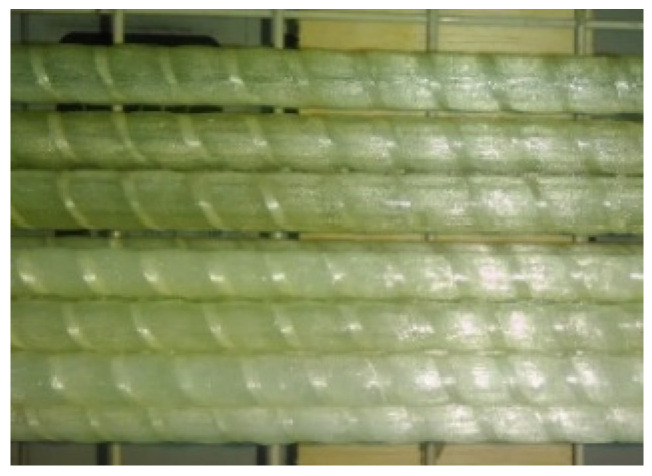
GFRP bars used in the experimental program.

Concerning the manufacturer, the GFRP bars used in the experimental program were produced with glass fibers resistant to alkaline environments and epoxy ester-vinyl resin, with a surface conformation characterized by helical grooves, as depicted in Figure 1, and presents tensile strength and an elasticity modulus of 1047 MPa and 48 GPa, respectively. According to NBR 17201-1 [51], GFRP or BFRP (Basalt Fiber-Reinforced Polymer) bars must meet the minimum mechanical properties presented in Table 4, among others. Additionally, the bars must be between the area limits of the cross-section to define the nominal diameter, as shown in Table 5.

**Figure 2:**
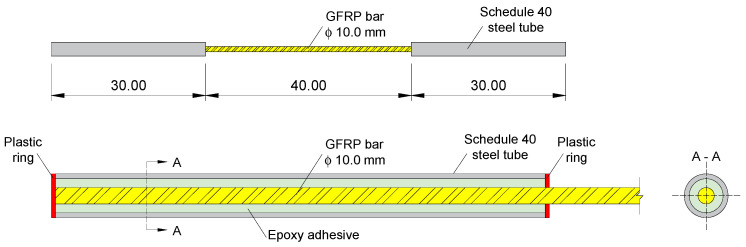
GFRP specimen scheme (units in centimeters).

The determination of effective diameter and effective area of the GFRP bars were performed in accordance with NBR 17201-2 [52]. Tensile tests were conducted on five specimens of GFRP bars with a length of 100 cm and a diameter of 10 mm for each exposure environment, following the ABNT NBR 17201-3 [53] procedures, to determine the stress–strain behavior and elasticity modulus. In consideration of the samples remaining protected, without contact with water or exposure to weather conditions, tensile tests were conducted on the reference specimens, and the results were extrapolated to the specimens maintained for 1000 h in the laboratory environment. The tensile tests of GFRP bars required steel anchors at each end of the specimen due to the low compressive strength of GFRP compared to its tensile strength, preventing GFRP crushing by the machine clamps. The steel anchors provide uniform pressure on the GFRP bars and avoid slippage as the tensile load increases [53]. Thus, Schedule 40 steel tubes with a length of 30 cm were used at each end, and the free length of the GFRP bar was 40 cm, as shown in Figure 2. The Sikadur 32 epoxy adhesive was used to bond the GFRP bar to the steel tube, and the alignment between GFRP bars and the steel tube was ensured using circular spacers made by 3D printing.

**Figure 3:**
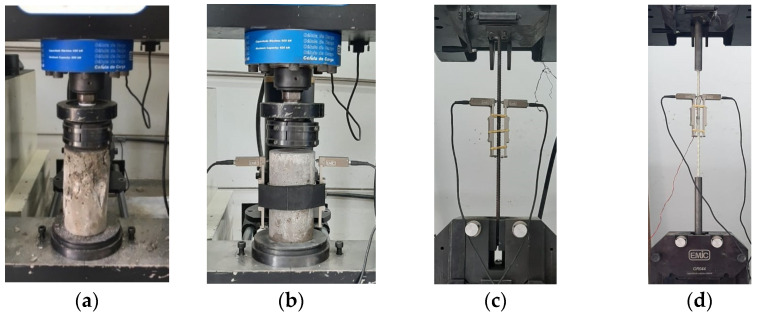
(**a**) Compressive strength and (**b**) elasticity modulus test in concrete specimens and tensile tests in (**c**) steel and (**d**) GFRP bars.

The fiber content was determined by combustion method according to ABNT NBR 17201-10 [54]. Figure 3 presents the materials characterization tests. All tests were conducted on an EMIC DL-60000 universal testing machine with a capacity of 600 kN, using a 150 mm electronic extensometer.

### 2.2. Reinforced Concrete Beams

**Figure 4:**
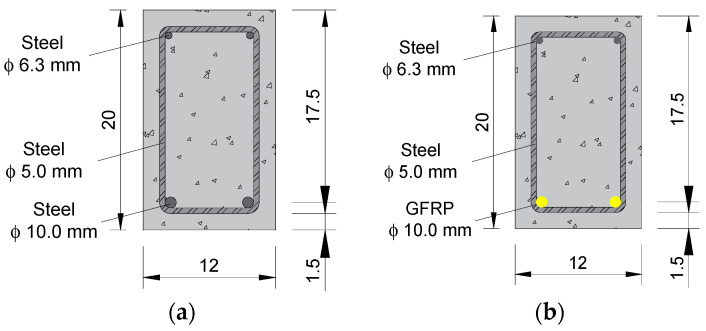
Cross-section of the beams reinforced with (**a**) steel bars and (**b**) GFRP bars—units in centimeters.

The twelve reinforced concrete beams had a cross-sectional dimension of 12 × 20 cm, an overall length of 250 cm, and a free span of 230 cm. The positive longitudinal reinforcement of the beams consisted of two steel or GFRP bars with a 10 mm diameter (longitudinal reinforcement rate of 0.75%). The negative longitudinal reinforcement was composed of two steel bars with a 6.3 mm diameter (to ensure the positioning of the stirrups), and the stirrups consisted of steel stirrups with a 5 mm diameter and a spacing of 10 cm, as shown in Figure 4. A concrete cover of 1.5 cm was used, smaller than that prescribed by most design codes for durability considerations. As such, chloride ingress was likely accelerated, amplifying the observed effects of exposure. This limitation means the results should be interpreted as a relative comparison between reinforcement types under severe accelerated conditions, rather than as direct field predictions for typical cover depths. The stirrups of the beams maintained under chloride attack were protected from corrosion using zinc-rich epoxy paint.

The steel and GFRP reinforced concrete beams were designed according to ACI 318 [47] and ACI 440.1R [30], respectively, to predict the nominal moment capacity and failure mode using the experimental data from the material testing results. Table 6 presents the nominal moment capacity (M_n_), the maximum resisted force (F_max_), and the expected failure mode for the beams.

According to Table 6, the design failure mode was a concrete crushing or balanced rupture, where the bar rupture and concrete crushing occur simultaneously. Concrete crushing is a brittle failure mode, but, as GFRP bars do not exhibit yielding like steel reinforcement, it is more desirable than GFRP rupture for this type of structure because there is still some ductility due to the development of the plastic behavior of concrete. If GFRP reinforcement fails, it can be catastrophic and occur without warning [6,16,30,55].

### 2.3. Exposure Environments

After testing the reference specimens, the remaining beams, concrete, steel, and GFRP specimens were exposed to two different environments: (a) a laboratory environment (internal and protected) and (b) a conditioning tank containing a NaCl solution with a concentration of 3.5% and a temperature of 50 ± 3 °C, following ACI 440.9R [31] adapted for the use of NaCl solution. These specimens remained in exposure environments for 1000 h.

**Figure 5:**
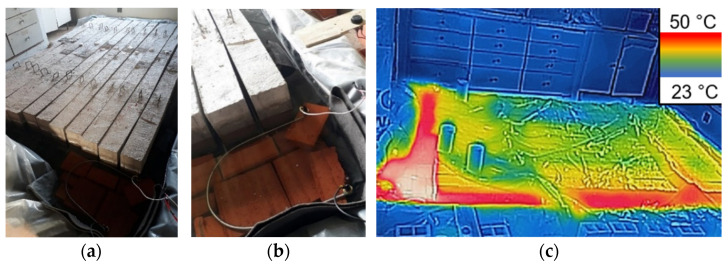
(**a**) Conditioning tank, (**b**) heating system and (**c**) thermographic image.

The conditioning tank was constructed in a protected environment using juxtaposed ceramic blocks, as shown in Figure 5a. The beams, concrete, steel, and GFRP specimens were positioned on ceramic blocks, allowing complete involvement, and the conditioning tank was filled with NaCl solution. The beams were immersed to a depth of 8 cm, and the conditioning tank was covered with a plastic cover.

For solution heating, a system consisting of an immersed resistance of 2000 W connected to a 30 A thermostat was used (Figure 5b). The solution temperature was periodically checked using a digital thermometer and thermographic images obtained with a FLIR Lepton camera, as shown in Figure 5c.

### 2.4. Instrumentation and Test Setup

Three-point bending tests were conducted to evaluate the flexural behavior of reinforced concrete beams after environmental exposure. The main parameters investigated included the load-deflection behavior, failure modes, crack pattern, and strains in concrete and longitudinal reinforcement.

**Figure 6:**
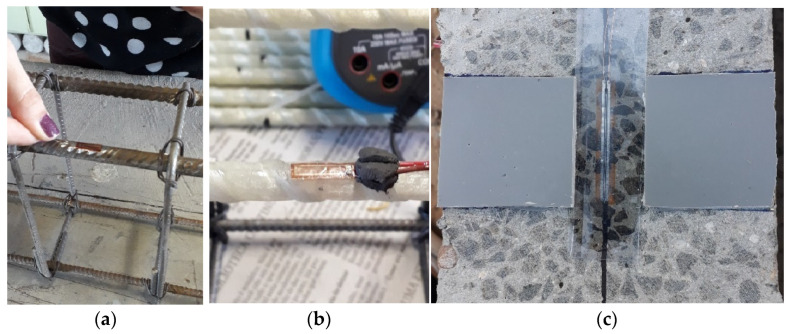
Strain gage attached on (**a**) steel bar, (**b**) GFRP bar, and (**c**) concrete surface.

The instrumentation of reinforced concrete beams involved using linear variable differential transformers (LVDT) and strain gauges (SG). Following the design of the reinforcements, the center of one steel and GFRP bar of the positive longitudinal reinforcement of each beam was prepared and strain gauges of 120 Ω and a reading grid length of 10 mm were attached to measure the strain along the test (Figure 6a,b). Strain gauges with a resistance of 120 Ω and a reading grid length of 50 mm were also bonded to the concrete surface to measure the concrete compressive strain (Figure 6c).

**Figure 7:**
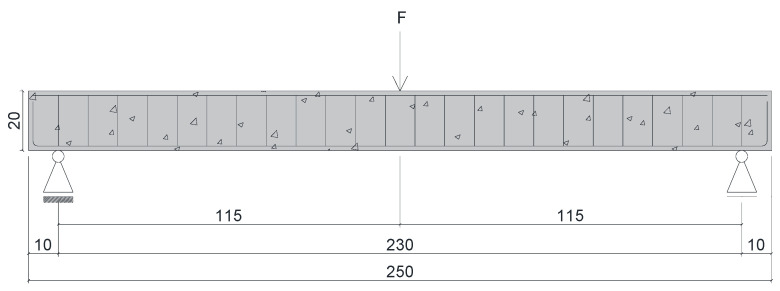
Three-point bending test setup—units in centimeters.

The three-point bending tests were conducted using an EMIC DL-60000 universal test machine with a maximum capacity load cell of 600 kN and read resolution of 0.1 kN. Displacement control was achieved through the internal transducer of the test machine with a rate of 1 mm/min, while the applied load was measured by the load cell (Figure 7).

**Figure 8:**
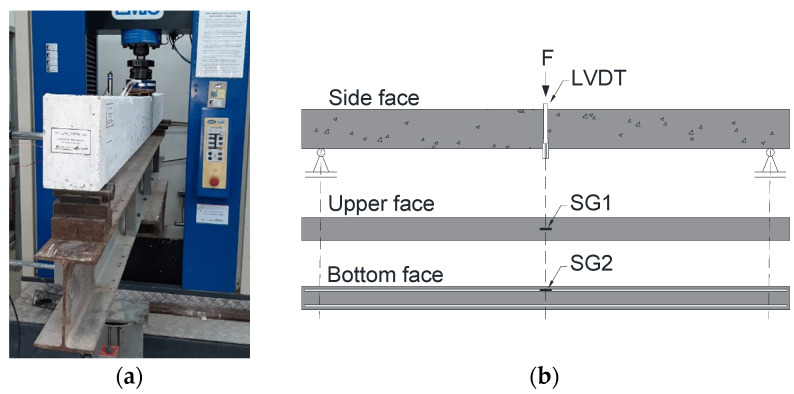
(**a**) Three-point bending test and (**b**) instrumentation of reinforced concrete beams.

The vertical displacement at the mid-span was measured using a Vishay LVDT with a reading field of 100 mm (±0.01 mm) fixed to an external support. Figure 8a presents the execution of the three-point bending test, and Figure 8b illustrates the instrumentation utilized, with SG1 and SG2 representing the strain gauges responsible for measuring concrete and steel/GFRP longitudinal reinforcement strains, respectively.

All test data was recorded in a data acquisition system.

### 2.5. Chloride Penetration Depth

**Figure 9:**
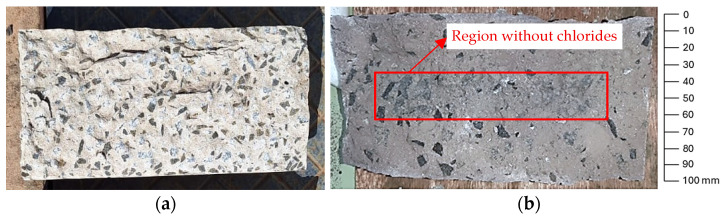
Concrete specimen submitted to a depth of chloride penetration test: freshly fractured concrete surface (**a**) and fractured surface to which silver nitrate has been applied (**b**).

The chloride penetration depth was assessed using the colorimetric method, which involves applying silver nitrate to the concrete surface to evaluate the extent of chloride attack. In the absence of chlorides, the concrete darkens, whereas in the presence of chlorides, the color remains clear, as shown in Figure 9. This figure illustrates a freshly fractured concrete surface (a) and a fractured surface to which silver nitrate has been applied (b). The central region of the concrete surface appears darker, indicating the area unaffected by chlorides. This test was performed to determine the chloride penetration depth in three concrete specimens exposed to a conditioning tank for 1000 h.

## 3. Results

### 3.1. Concrete, Steel, and GFRP

Table 7 presents the results obtained in materials characterization, where f_cm_ and E_cm_ correspond to the compressive strength and elasticity modulus of concrete, f_y_, e_sy_, and E_s_ correspond to the yield strength, yield strain, and elasticity modulus of steel, respectively, and f_fu_, e_fu_, and E_f_ correspond to the tensile strength, rupture strain, and elasticity modulus of GFRP. The coefficients of variation reported in Table 7 ranged from 1 to 10%. Thus, the relatively low COVs confirm the reliability of the experimental results and reduce concerns regarding variability from specimen preparation or testing.

**Figure 10:**
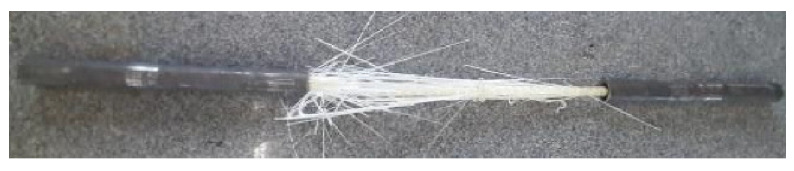
Failure of GFRP specimen subjected to tensile test.

Figure 10 depicts the failure mode of GFRP bars subjected to tensile test. It occurred along the free length of the specimen and was attributed to the rupture of glass fibers, where splitting was observed.

**Figure 11:**
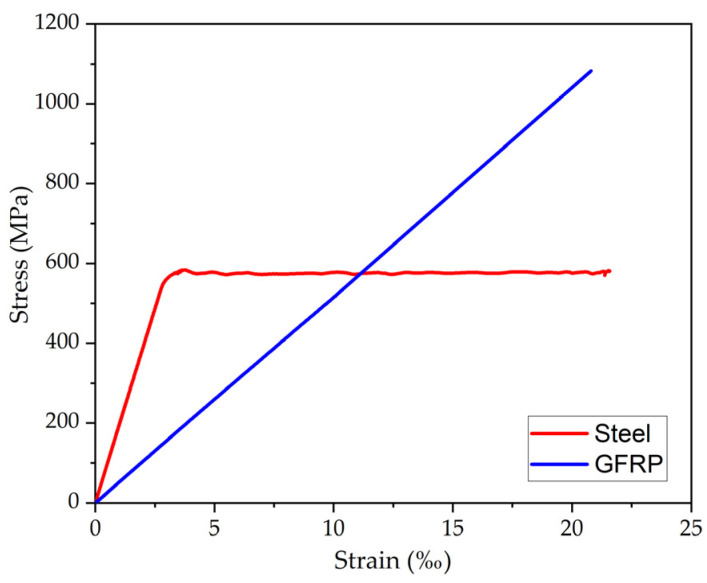
Average stress–strain curves of reference steel and GFRP specimens subjected to tensile test.

Figure 11 presents the average stress–strain curves of reference steel and GFRP bars. While steel bars exhibit a clear yielding condition, indicative of ductile behavior, GFRP bars demonstrate linear behavior until failure, with a brittle rupture. The difference in the slopes of the curves highlights the disparity in the modulus of elasticity between the materials, considering that the modulus of elasticity of steel is approximately four times higher than that of GFRP. Additionally, it is observed that the tensile strength in GFRP is approximately two times higher than that of steel.

Concerning the GFRP bar diameter, an average effective diameter of 9.44 mm, leading to an average effective area of 69.98 mm^2^, was obtained. Comparing the average effective area obtained with the limits presented in ABNT NBR 17201-1 [51] (available in Table 5), it is noted that this value falls within the range of 47.2 to 70.7 mm^2^. Therefore, the nominal diameter of the GFRP bars used as reinforcement in the concrete beams is 8 mm.

Finally, for the fiber content, an average value of 77.2% was obtained, higher than 75% specified by ABNT NBR 17201-1 [51].

### 3.2. Chloride Ions Penetration Depth

**Figure 12:**
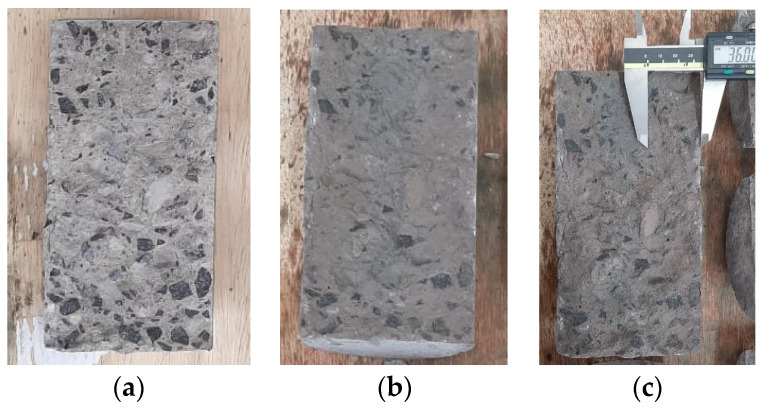
Concrete specimens (**a**) before and (**b**) after the application of silver nitrate spray, and (**c**) measurement of chloride penetration depth.

The chloride penetration depth test was conducted on concrete specimens subjected to a conditioning tank for 1000 h. For this purpose, three concrete specimens were fractured by diametrical compression tensile, and a silver nitrate solution was sprayed on the concrete surface. The chloride penetration depth was then quantified using a digital pachymeter, as depicted in Figure 12. The obtained depth values were 34.89 mm, 36.42 mm, and 36.91 mm for each specimen. These values significantly exceed the concrete cover thickness of 15 mm, providing clear evidence that chlorides reached the longitudinal reinforcements.

### 3.3. Reinforced Concrete Beams

Table 8 provides a summary of the results obtained for each beam, where F denotes the recorded applied load (kN), δ represents the vertical mid-span displacement (mm), ε_s/f_ is the reinforcement strain (‰), and ε_c_ denotes the concrete strain (‰). These results were assessed at the cracking initiation, yielding of steel reinforcement, concrete crushing, and when the maximum load was obtained.

A specific stop criterion was not established during the testing of reference concrete beams reinforced with steel bars (V_LAB_S_45d). Subsequent tests on reference concrete beams reinforced with GFRP bars (V_LAB_GFRP_45d) were conducted until the longitudinal reinforcement rupture revealed displacements of approximately 70 mm. Consequently, for the remaining steel-reinforced beams (V_LAB_S_1000h and V_ACP_S_1000h), the test stopped when the beams reached a displacement of 75 mm. In the case of the remaining GFRP-reinforced concrete beams (V_LAB_GFRP_1000h and V_ACP_GFRP_1000h), the stop criterion remained the rupture of longitudinal reinforcement. It was noted that some strain gauges suffered damage due to the presence of water (V2_LAB_S_1000h, V1_ACP_GFRP_1000h, and V2_ACP_GFRP_1000h) or due to the development of concrete cracks in the positive region.

It can be observed in Table 8 that, at the onset of yielding in the steel reinforcement, tensile strains in the longitudinal reinforcement ranged between 2.1‰ and 2.4‰, while compressive strains in the concrete were approximately 1.0‰. Concerning the initiation of concrete crushing, concrete strains of around 3.0‰ were recorded, similar to the concrete crushing value indicated on ACI 318 [47] and ACI 440.1R [30].

**Figure 13:**
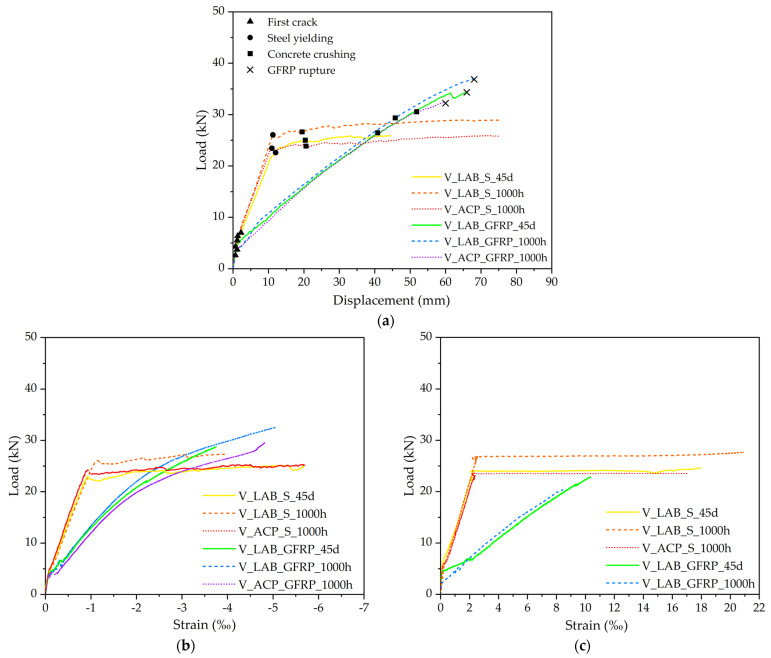
Load versus vertical displacement relationship (**a**), Load versus strains in concrete (**b**) and Load versus strains in longitudinal reinforcements (**c**).

Figure 13a presents the average Load vs. displacement relationship of the beams, and Figure 13b,c present the average Load vs. strains in concrete and longitudinal reinforcements.

Figure 13a analysis reveals a typical behavior of concrete beams reinforced with steel bars, delineated by three stages. The initial stage represents uncracked concrete, exhibiting linear elastic behavior. The second stage depicts cracked concrete with steel in the elastic regime, followed by a reduction in the element stiffness. The final stage includes the cracked concrete with steel yielding, presenting a well-defined yield level. In contrast, concrete beams reinforced with GFRP bars exhibit behavior characterized by two stages. The first stage corresponds to uncracked concrete, showing a linear elastic behavior. The second stage illustrates cracked concrete and GFRP with an approximately linear behavior until rupture.

In the pre-cracking phase, all beams exhibited similar behavior characterized by high bending stiffness, as the concrete works in the elastic regime, effectively resisting the applied loads. However, post-cracking, there was a transition where the reinforcement started to resist the applied loads. This transition was evident in the altered inclination of the curves, showing a loss of bending stiffness in the structural elements. The reduction in bending stiffness from pre- to post-cracking was approximately 49%, 59%, and 60% for beams V_LAB_S_45d, V_LAB_S_1000h, and V_ACP_S_1000h, respectively. Notably, concrete beams reinforced with GFRP bars exhibited a more significant decrease in bending stiffness of approximately 76% due to the lower elasticity modulus of GFRP. Specifically, the reduction in bending stiffness from pre- to post-cracking was approximately 91%, 86%, and 81% for beams V_LAB_GFRP_45d, V_LAB_GFRP_1000h, and V_ACP_GFRP_1000h, respectively. Comparatively, the bending stiffness of V_LAB_GFRP_45d, V_LAB_GFRP_1000h, and V_ACP_GFRP_1000h was 66%, 72%, and 70% lower than that of V_LAB_S_45d, V_LAB_S_1000h, and V_ACP_S_1000h, respectively. These results underscore the substantial impact of GFRP reinforcement on the post-cracking bending stiffness of concrete beams, owing to the inherent characteristics of GFRP bars, such as their lower elasticity modulus.

The lower elasticity modulus of GFRP bars also impacted the displacements observed in GFRP-reinforced concrete beams, resulting in higher vertical displacements than steel-reinforced concrete beams. Concerning concrete crushing, the vertical displacements of the GFRP-reinforced concrete beams were approximately 78% greater than those of steel-reinforced concrete beams. Despite the larger displacements, GFRP-reinforced concrete beams exhibited superior loading-carrying capacity than steel-reinforced concrete beams. The maximum attained applied loads of the V_LAB_GFRP_45d, V_LAB_GFRP_1000h, and V_ACP_GRFRP_1000h beams were 37%, 27%, and 28% higher than those of the V_LAB_S_45d, V_LAB_S_1000h, and V_ACP_S_1000h beams, respectively. This difference can be attributed to the high tensile strength of GFRP bars.

In the context of exposure environments, the V_ACP_S_1000h beams exhibited maximum loading-carrying capacity 12% lower than the V_LAB_S_1000h beams while exhibiting no disparity in stiffness. This discrepancy is due to the degradation of steel reinforcement induced by corrosion. The V_ACP_GFRP_1000h and V_LAB_GFRP_1000h beams demonstrated comparable stiffness, yet the former experienced fracture with force and displacement values approximately 10% and 8% lower than the V_LAB_GFRP_1000h beams. This discrepancy can be attributed to the water absorption by GFRP bars.

The beam-level performance observed in this study aligns well with existing durability benchmarks for GFRP reinforcement. Robert, Cousin and Benmokrane [42] and Robert and Benmokrane [43] demonstrated that chloride and moist concrete exposures reduce bar strength and bond, effects that were reflected at the structural scale in the ≈10% reduction in GFRP-RC beam capacity. Similarly, Benmokrane et al. [22] reported strength retention between 85 and 95% for epoxy-based GFRP bars after 1000 h saline/alkaline conditioning; the ≈90% capacity retention observed in present beams is quantitatively consistent with this range.

Figure 13b,c illustrate the distinctions in the applied load-strain curves of concrete and longitudinal reinforcement, presenting analogous stages found in the applied load–displacement curves of reinforced concrete beams. In the post-cracking elastic stage, for the same load level, the concrete in beams reinforced with GFRP bars exhibits deformations approximately twice as large (Figure 13b). For the steel-reinforced beams, a plateau is observed, whereas the GFRP-reinforced beams show an approximately linear response, with deviation from linearity occurring beyond strains of about 2‰, corresponding to the onset of the plastic behavior of concrete. With respect to Figure 13c, a plateau can be observed in the load–strain curves of the steel-reinforced beams, while the GFRP-reinforced beams display a linear behavior up to failure, similar to that observed in tensile tests. In the post-cracking elastic stage, deformations about three times larger are observed.

**Figure 14:**
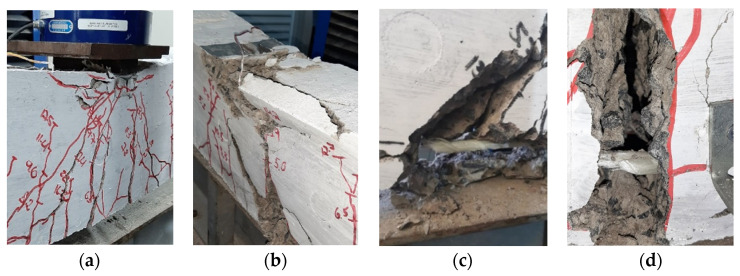
Failure modes of the beams: (**a**) concrete crushing of the steel reinforced concrete beams, (**b**) concrete crushing of the GFRP reinforced concrete beams, and (**c**,**d**) rupture of GFRP bars.

**Figure 15:**
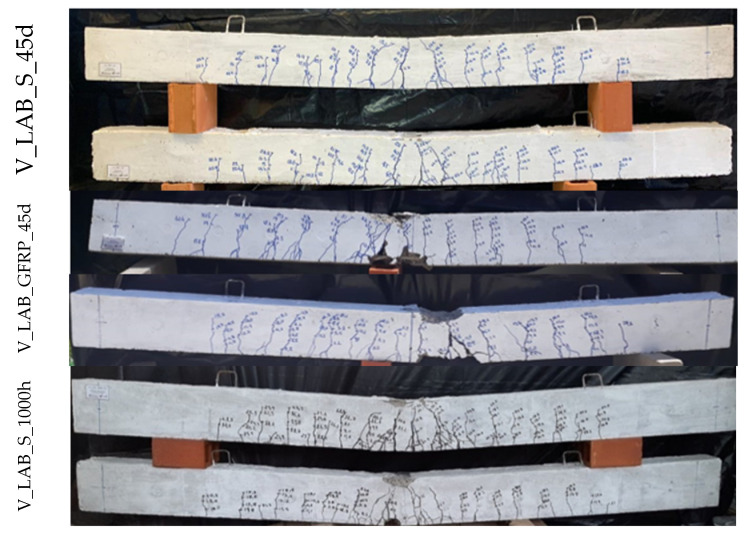

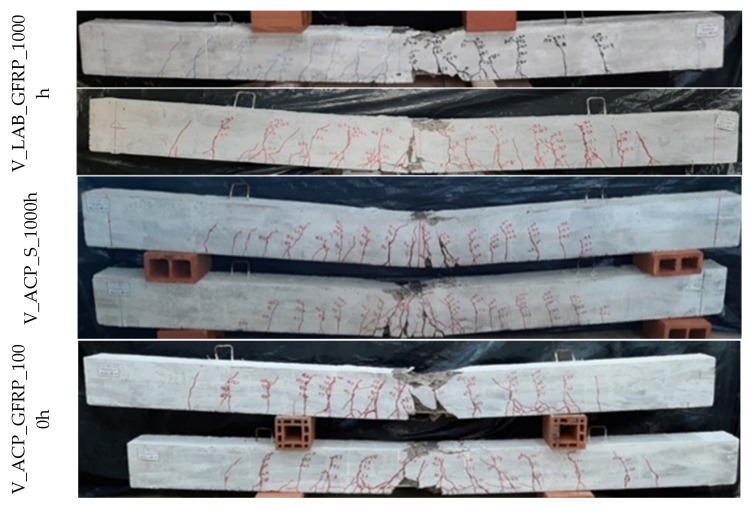
The crack pattern of the beams.

The failure mode observed in steel-reinforced concrete beams was ductile and characterized by steel yielding followed by concrete crushing. In contrast, GFRP-reinforced concrete beams exhibited a failure mode involving concrete crushing followed by GFRP rupture, representing a brittle failure mode. Figure 14 presents the failure mode of the beams and Figure 15 illustrates the crack pattern of the beams.

Initially, the cracks exhibited a vertical orientation at the mid-span of the beams due to bending. As the applied load increased, the cracks assumed an inclined trajectory while propagating towards the supports, owing to the combined influence of bending and shear. These cracks extended vertically along the height of the beams, progressing towards the compression zone. Due to the low modulus of elasticity of GFRP bars, concrete beams with GFRP bars experienced higher deflections and larger crack widths when compared to concrete beams reinforced with steel bars [16,56]. Beams V_LAB_GFRP_45d, V_LAB_GFRP_1000h, and V_ACP_GFRP_1000h exhibited first-crack initiation loads that were 39%, 59%, and 33% lower, respectively, than those of beams V_LAB_S_45d, V_LAB_S_1000h, and V_ACP_S_1000h (Table 4). In this way, it was observed that the GFRP-reinforced concrete beams manifested cracks characterized by greater width and length, attributable to the lower elasticity modulus of GFRP. This lower modulus induces a shallower neutral axis depth and a reduced compression region.

Concerning degradation, Table 8 shows that beams V_ACP_S_1000h and V_ACP_GFRP_1000h exhibited reductions of 21% and 14% in the first-cracking load compared to beams V_LAB_S_45d and V_LAB_GFRP_45d, respectively. However, no changes were observed in the crack pattern because of degradation, which is consistent with the preservation of the post-cracking stiffness of the beams.

Although macroscopic observations of failure were detailed, no microscopic evidence was obtained. Mechanistic insight into fiber–matrix degradation, such as fiber pull-out, interfacial debonding, or resin plasticization, was not directly observed. These processes likely contributed to the observed stiffness losses and crack widening in GFRP-RC beams. Future research should include Scanning Electron Microscopy (SEM) or other microstructural techniques to validate the hypothesized mechanisms.

Table 9 presents a comparative analysis of failure modes and maximum forces acquired using the ACI design codes (F_max,d_) and the failure modes and maximum forces obtained in experimental tests (F_max,e_).

Across all beams, the ratio of design to experimental maximum forces was less than one, signifying that the design results were lower than experimental outcomes. Therefore, the design results exhibited a conservative tendency, and the predictions leaned towards safety, and all the ACI design codes continued to demonstrate accurate predictions of the failure modes observed experimentally.

The beams reinforced with steel bars showed closer agreement with the experimental values (average ratio equals to 0.92) compared to those reinforced with GFRP bars. The experimental maximum loads of GFRP-RC beams were 20–30% higher than ACI 440.1R predictions [30], with design-to-experiment ratios of 0.72–0.81. This conservative bias is expected, as ACI provisions adopt reduced effective strain limits and simplified compression block assumptions to ensure safety. While this leads to underestimation of moment capacity, it confirms that current design rules remain conservative even after chloride conditioning. Future refinements to ACI 440.1R [30] could improve prediction accuracy without compromising safety by better accounting for FRP tensile behavior and bond mechanisms.

The higher experimental strengths of GFRP-RC beams compared to ACI 440.1R [30] predictions can be attributed to several conservative assumptions: (i) reduced effective strain limits imposed to avoid brittle rupture, (ii) simplified rectangular stress block parameters that underestimate compression capacity, and (iii) omission of potential beneficial bond mechanisms. While this ensures safety, it also indicates that serviceability rather than ultimate strength governs the design of GFRP-RC members.

## 4. Conclusions

The primary objective of this study was to evaluate the application of accelerated conditioning protocols described in ACI 440.9R [31] on concrete beams reinforced with steel and GFRP bars. The use of GFRP bars is justified by their superior resistance to corrosion, which often causes deterioration in conventional steel bars. However, they can undergo degradation due to moisture, alkalinity, and temperature, among other factors. In this context, an experimental program involving three-point bending tests on reinforced concrete beams reinforced with steel and GFRP bars was carried out. These beams were subjected to laboratory conditions and a conditioning tank containing a NaCl solution with a concentration of 3.5%, maintained at 50 ± 3 °C for 1000 h.

Thus, the conclusions of this study are outlined below:

- Until the occurrence of cracking, all beams displayed similar behavior, with the concrete effectively withstanding applied loads. Following the cracking initiation, the reinforcements came into play, leading to a noticeable loss of stiffness. The reductions in bending stiffness from pre- to post-cracking were approximately 49%, 59%, and 60% for beams V_LAB_S_45d, V_LAB_S_1000h, and V_ACP_S_1000h, respectively. Additionally, the reductions were about 91%, 86%, and 81% for beams V_LAB_GFRP_45d, V_LAB_GFRP_1000h, and V_ACP_GFRP_1000h, respectively. The more pronounced decreases in bending stiffness observed in GFRP-reinforced concrete beams from pre- to post-cracking can be attributed to the lower elasticity modulus of GFRP, approximately 76% lower than the elasticity modulus of steel bars. Consequently, the bending stiffness from pre- to post-cracking for beams V_LAB_GFRP_45d, V_LAB_GFRP_1000h, and V_ACP_GFRP_1000h were 66%, 72%, and 70% lower than those for V_LAB_S_45d, V_LAB_S_1000h, and V_ACP_S_1000h, respectively.
- The reduced elasticity modulus of GFRP bars also influences the displacements of GFRP-reinforced concrete beams, resulting in vertical displacements at mid-span approximately 78% greater than those observed in steel-reinforced concrete beams. Conversely, GFRP-reinforced concrete beams exhibit an increased load capacity, with the maximum resisted force of beams V_LAB_GFRP_45d, V_LAB_GFRP_1000h, and V_ACP_GFRP_1000h exceeding that of beams V_LAB_S_45d, V_LAB_S_1000h, and V_ACP_S_1000h by 37%, 27%, and 28%, respectively.
- The exposure of steel-reinforced concrete beams to a chloride solution for 1000 h resulted in a maximum resisted load 12% lower than that observed in steel-reinforced concrete beams subjected to a laboratory environment for the same duration, indicating steel degradation. Concerning GFRP reinforcement, the GFRP-reinforced concrete beams exposed to a chloride solution for 1000 h exhibited a failure force and displacement approximately 10% and 8% lower, respectively, than that GFRP-reinforced concrete beams maintained in a laboratory environment for the same duration, indicating degradation of the GFRP.
- The steel-reinforced concrete beams exhibited a ductile failure mode, characterized by steel yielding followed by concrete crushing, while the GFRP-reinforced concrete beams displayed a brittle failure mode, marked by concrete crushing followed by GFRP rupture.
- The diminished elasticity modulus of GFRP influences the crack pattern of GFRP-reinforced concrete beams, as these beams exhibit cracks with greater height and length than steel-reinforced concrete beams.
- The guidelines of ACI design codes correctly predicted the failure modes of steel and GFRP-reinforced concrete beams and contributed to safety, given that the design results were conservative compared to the experimental results.
- Concerning the engineering interpretation, the findings of this study indicate that GFRP-RC beams exposed to chloride environments under ACP conditions retain sufficient flexural strength to meet the conservative predictions of ACI 440.1R for ultimate limit states.

Unlike previous durability studies limited to bar-level ACPs, this work demonstrated for the first time how ACI 440.9R protocols translate to structural beam performance, thus bridging the gap between material-level durability and structural-scale behavior. Thus, it can be concluded that beams subjected to a chloride solution underwent degradation regardless of the reinforcement material. Steel-reinforced concrete beams experienced a loss in load capacity due to steel corrosion, whereas GFRP-reinforced concrete beams exhibited a reduction in load capacity due to the degradation of GFRP bars caused by water. Therefore, accelerated conditioning protocols are also viable for application to structural elements rather than solely to FRP bars.

However, the present investigation was limited to (i) a single exposure duration (1000 h), (ii) a reduced concrete cover depth of 15 mm, (iii) a single chloride concentration (3.5% NaCl), and (iv) monotonic flexural loading. While these choices ensured sensitivity to degradation, they do not fully replicate field conditions. Future research should extend to longer exposures, cyclic and sustained loading, variable chloride concentrations, and beams with standard cover depths. Microstructural analyses (e.g., SEM) are also required to confirm the fiber–matrix degradation mechanisms suggested by the present macroscopic observations.

From an engineering standpoint, these findings confirm that GFRP-RC beams maintain adequate safety margins even under aggressive chloride conditioning. The conservatism of ACI 440.1R predictions was verified at the structural level, suggesting that existing design provisions remain reliable. However, refinement of design models, particularly regarding bond behavior and effective strain assumptions, could improve accuracy without compromising safety.

## Figures and Tables

**Table 1:** Previous test results of GFRP bars in concrete.

Reference	Fiber	Resin	Surface	Environmental Exposure	Temperature (°C)	Time (Days)	Tensile Strength (MPa)	Retention (%)
Chen et al. [32]	E-glass	Vinyl ester	Helically wrapped and slightly sand coated	Unconditioned	-	-	771	100
				Tap water immersion	60	70	544	71
				Alkaline solution immersion	60	70	564	73
				Saline solution immersion	60	70	572	74
	E-glass	Vinyl ester	Helically wrapped and slightly sand coated	Unconditioned	-	-	925	100
				Alkaline solution immersion	60	60	482	52
				Embedded in concrete and immersed in tap water	Room	90	836	90
				Embedded in concrete and immersed in alkaline solution	60	90	566	61
Robert, Cousin and Benmokrane [42]	E-glass	Vinyl ester	Sand coated	Unconditioned	-	-	786	100
				Embedded in concrete and immersed in tap water	23	240	714	91
					40	240	708	90
					50	240	665	85
Robert and Benmokrane [43]	E-glass	Vinyl ester	Sand coated	Unconditioned	-	-	786	100
				Embedded in concrete and immersed in saline solution	23	365	726	92
					40	365	712	91
					50	365	702	89
					70	120	744	95
Almusallam et al. [44]	E-glass	Vinyl ester	Regular ribs	Unconditioned	-	-	1478	100
				Embedded in concrete and immersed in tap water	50	180	1229	83
					50	360	1158	78
					50	540	1123	76
				Embedded in concrete and immersed in seawater	50	180	1269	86
					50	360	1300	88
					50	540	1238	84
Fergani et al. [2]	ECR glass	Vinyl ester	Ribs	Unconditioned	-	-	1542	100
				Embedded in concrete and immersed in tap water	60	42	1244	81
					60	83	1227	80
					60	270	917	59
Jia et al. [4]	E-glass	Vinyl ester	Helically wrapped and sand coated	Unconditioned	-	-	-	100
				Embedded in concrete and immersed in tap water	60	120	-	61.6
				Embedded in concrete and immersed in salt water	60	120	-	60.7
Morales et al. [26]	ECR glass	Vinyl ester	Double helically fiber-wrapped	Unconditioned	-	-	822	100
				Embedded in seawater concrete and exposed to subtropical region	25	30	821	100
						180	795	97
						360	827	101
						720	811	99
				Embedded in seawater concrete and immersed in seawater	60	30	764	93
						180	679	83
						360	616	75
						720	607	74
Wang et al. [46]	Glass	Vinyl ester	Ribs and sand coated	Unconditioned	-	-	1102	100
				Embedded in seawater and sea sand concrete and subjected to seawater wet-dry cycles	40 (cycles)	90	-	81
						180	-	76
						270	-	76

**Table 2:** Brief resume of some experimental program.

Researchers	Environment	Exposure	Evaluation	Structural Beams Tested?
Robert, Cousin and Benmokrane [42]	Bars in moist concrete	Tap water/pore solution, 23–50 °C	Bar property retention	No
Robert and Benmokrane [43]	Concrete-wrapped bars	Saline + moist concrete	Residual bar properties	No
Benmokrane et al. [22]	Bars (different resins)	Alkaline solution, 60 °C	Durability by resin type	No
Fergani et al. [2]	Bars/coupons under stress	Severe environments + sustained load	Degradation mechanisms	No
This study	Full RC beams (steel & GFRP)	ACI 440.9R NaCl 3.5%, 50 °C, 1000 h	Flexural stiffness, cracking, failure, design conservatism	Yes

**Table 3:** Overview of the experimental program.

Exposure Environment	Age of Test	Specimens				
		Beams		Concrete	Steel	GFRP
		Steel	GFRP			
Laboratory	45 days	2	2	3	2	5
Laboratory	45 days + 1000 h (87 days)	2	2	3	2	
Chloride attack	45 days + 1000 h (87 days)	2	2	3	2	5

**Table 4:** Minimum values of the mechanical properties of the bars.

Properties	Characteristic Values of Mechanical Properties for GFRP and BFRP Members
Fiber content (%)	≥75
Tensile strength (MPa)	≥800
Modulus of elasticity (GPa)	≥45
Ultimate tensile strain (%)	≥1.1
Effective cross-section	Table 5

**Table 5:** Diameters and nominal areas of fiber-reinforced bars.

Nominal Diameter (mm)	Nominal Cross-Section Area (mm^2^)	Effective Area Limits of the Cross-Section (mm^2^)	
		Minimum	Maximum
4	12.6	11.8	17.7
5	19.6	18.5	25.4
6	28.3	26.6	45.2
8	50.3	47.2	70.7
10	78.5	73.8	101.8
12	113.1	106.3	138.5
14	153.9	144.7	181.0
16	201.1	189.0	229.0
18	254.5	239.2	282.7
20	314.2	295.3	342.1
22	380.1	357.3	441.8
25	490.9	461.4	554.2
28	615.7	578.8	723.8
32	804.2	756.0	910.0

Note: Table 1 of the NBR 17201-1 [51].

**Table 6:** Expected flexural behavior of the beams.

Beam	Design Code	M_n_ (kN·m)	F_max_ (kN)	Failure Mode
V_LAB_S_45d	ACI 318 [47]	14.36	24.98	Steel yielding
V_LAB_S_1000h		14.34	24.94	Steel yielding
V_ACP_S_1000h		14.20	24.70	Steel yielding
V_LAB_GFRP_45d	ACI 440.1R [30]	15.28	26.57	Concrete crushing
V_LAB_ GFRP _1000h		15.42	26.82	Concrete crushing
V_ACP_ GFRP _1000h		15.40	26.79	Balanced rupture

**Table 7:** Experimental results of the materials characterization.

Exposure Condition	Age (Days)	Concrete		Steel			GFRP		
		f_cm_ (MPa)	E_cm_ (GPa)	f_y_ (MPa)	ε_sy_ (‰)	E_s_ (GPa)	f_fu_ (MPa)	ε_fu_ (‰)	E_f_ (GPa)
Laboratory	45	32.79 (10.21)	- ^1^	565.35 (0.08)	3.07 (4.69)	194.33 (1.16)	1048.98 (4.90)	20.83 (3.96)	50.36 (2.00)
Laboratory	87	33.49 (2.00)	34.50 (1.17)	563.36 (0.06)	3.02 (7.65)	197.47 (0.67)			
Chloride attack	87	34.60 (1.08)	37.84 (10.63)	559.85 (0.06)	2.99 (2.97)	195.80 (1.35)	911.86 (4.73)	17.89 (3.13)	50.96 (2.10)

^1^ It was not possible to obtain E_cm_ at this age due to issues with the testing machine.

**Table 8:** Experimental results of the reinforced concrete beams.

Beams	First Crack				Reinforcement Yielding				Concrete Crushing				Maximum Load			
	F	δ	ε_s/f_	ε_c_	F	δ	ε_s/f_	ε_c_	F	δ	ε_s/f_	ε_c_	F	δ	ε_s/f_	ε_c_
	kN	mm	‰	‰	kN	mm	‰	‰	kN	mm	‰	‰	kN	mm	‰	‰
V1_LAB_S_45d	7.1	1.9	0.2	−0.2	23.0	10.9	2.1	−0.9	24.0	19.6	13.9	−2.6	25.4	49.2	x	x
V2_LAB_S_45d	6.9	2.6	x	x	24.7	13.3	x	x	26.0	21.2	x	x	26.7	32.9	x	x
V1_LAB_GFRP_45d	4.8	1.1	0.1	−0.1	-	-	-	-	27.3	40.2	x	−3.2	38.1	70.1	x	−4.6
V2_LAB_GFRP_45d	3.8	0.3	0.1	−0.1	-	-	-	-	25.6	41.5	x	−3.1	33.1	61.5	x	x
V1_LAB_S_1000h	6.3	1.2	0.3	−0.1	25.7	12.1	2.4	−1.3	26.9	20.7	8.4	−3.2	28.6	61.7	x	x
V2_LAB_S_1000h	6.4	1.4	x	−0.2	26.0	11.3	x	−1.2	27.7	18.3	x	−3.1	29.6	74.6	x	x
V1_LAB_GFRP_1000h	4.1	0.7	0.1	−0.1	-	-	-	-	28.5	44.7	x	−3.5	37.2	67.8	x	−6.4
V2_LAB_GFRP_1000h	1.1	0.6	0.3	−0.1	-	-	-	-	30.2	46.9	x	x	36.8	67.7	x	x
V1_ACP_S_1000h	5.1	1.2	0.1	−0.1	22.7	10.6	2.3	x	23.2	20.8	x	x	25.1	73.1	x	x
V2_ACP_S_1000h	5.9	1.2	0.4	−0.1	24.2	11.3	2.3	−0.9	24.5	20.4	x	−3.2	26.8	74.8	x	x
V1_ACP_GFRP_1000h	3.4	1.0	x	−0.1	-	-	-	-	29.1	49.7	x	x	31.7	59.3	x	x
V2_ACP_GFRP_1000h	4.0	1.3	x	−0.1	-	-	-	-	32.0	54.0	x	x	34.5	65.2	x	x

x—strain gauge damaged.

**Table 9:** Comparative analysis of design and experimental results.

Beam	Design		Experimental		F_max,d_/F_max,e_
	F_max,d_ (kN)	Failure Mode	F_max,e_ (kN)	Failure Mode	
V_LAB_S_45d	24.98	Steel yielding	26.05	Steel yielding	0.96
V_LAB_S_1000h	24.94	Steel yielding	29.10	Steel yielding	0.86
V_ACP_S_1000h	24.70	Steel yielding	25.95	Steel yielding	0.95
V_LAB_GFRP_45d	26.57	Concrete crushing	35.60	Concrete crushing	0.75
V_LAB_GFRP_1000h	26.82	Concrete crushing	37.00	Concrete crushing	0.72
V_ACP_GFRP_1000h	26.79	Balanced rupture	33.10	Concrete crushing	0.81

## Data Availability

The datasets presented in this article are not readily available because the data are part of an ongoing study. Requests to access the datasets should be directed to glaucia.dalfre@ufscar.br.
